# Reliability and utility of the Toronto Upper Gastrointestinal Cleaning Score for optimizing mucosal visualization during esophagogastroduodenoscopy

**DOI:** 10.1055/a-2781-6570

**Published:** 2026-04-09

**Authors:** Alfonso Fernandez-Ramirez, David Aguirre-Villarreal, Ivonne Hurtado-Diaz de Leon, Erick Jasso-Baltazar, Arturo Navarro-Sánchez, Jessica Paola Guerrero-Cabrera, Hiram Noel Tadeo-Espinoza, Guillermo Rosales-Sotomayor, Luis Carlos Chávez García, Berenice M Román-Calleja, Ricardo Ulises Macías-Rodríguez, Sergio A. Sánchez-Luna, Rafael Barreto-Zuñiga

**Affiliations:** 1 Endoscopy, Instituto Nacional de Ciencias Medicas y Nutrición Salvador Zubiran, Mexico City, Mexico; 2 Gastroenterology, Instituto Nacional de Ciencias Medicas y Nutrición Salvador Zubiran, Mexico City, Mexico; 3 6596 Division of Gastroenterology, Hepatology, and Nutrition, Allegheny Health Network, Pittsburgh, United States; 4 Instituto Mexicano de Cirugía Endoscópica y Bariátrica (IMCEB), Guadalajara, Mexico; 5 9967 Basil I. Hirschowitz Endoscopic Center of Excellence, The University of Alabama at Birmingham Heersink School of Medicine, Birmingham, United States

**Keywords:** Endoscopy Upper GI Tract, Precancerous conditions & cancerous lesions (displasia and cancer) stomach, Diagnosis and imaging (in chromoendoscopy, NBI, iSCAN, FICE, CLE)

## Abstract

**Background and study aims:**

Esophagogastroduodenoscopy (EGD) is a key tool for diagnosing upper gastrointestinal lesions, but its accuracy relies on optimal mucosal visualization. Currently, there are few validated instruments to objectively assess mucosal cleanliness. This study aimed to validate the Toronto Upper Gastrointestinal Cleaning Score (TUGCS) and explore its association with lesion detection.

**Patients and methods:**

In this prospective, single-center study, 175 patients undergoing diagnostic EGD were enrolled. Interobserver and intraobserver reliability of the TUGCS was assessed using intraclass correlation coefficients (ICCs) among expert and non-expert endoscopists. Secondary outcomes included detection of endoscopic lesions, which were analyzed using multivariate logistic regression adjusted for age, procedure duration, and indication. Additional analyses included Spearman’s correlation and z-tests.

**Results:**

TUGCS demonstrated excellent interobserver reliability (ICC = 0.95; 95% confidence interval 0.93–0.96) and intra-observer agreement (ICC = 0.896 for non-experts, 0.924 for experts). The score correlated strongly with subjective assessments ( *P* = 0.68; *P* < 0.001). Both TUGCS as a continuous variable and TUGCS ≥ 6 were independently associated with increased detection of gastroduodenal findings as a composite outcome, when adjusted for age, sex, simethicone use, and procedure duration.

**Conclusions:**

TUGCS is a reliable and reproducible tool across varying levels of endoscopic expertise. Higher scores were associated with improved detection of gastroduodenal pathology, supporting its utility as a quality metric in EGD. Multicenter studies are warranted to validate its role in broader clinical practice.

## Introduction

Esophagogastroduodenoscopy (EGD) is an essential diagnostic tool for evaluating the upper gastrointestinal tract. It is widely available and offers ongoing technological advancements, such as high-definition imaging and virtual chromoendoscopy. Despite these innovations, the diagnostic yield of EGD remains heavily dependent on adequate mucosal visualization because suboptimal cleaning can lead to missed lesions. Evidence suggests that as many as 10% to 11.3% of upper gastrointestinal cancers are missed during EGDs, delaying diagnoses and potentially leading to worse patient outcomes 1 2 . In Western countries, where gastric cancer incidence remains a public health concern, improving EGD quality is crucial for earlier detection and improved survival 3 .

The importance of mucosal visualization as a quality metric is well-established in gastrointestinal endoscopy. For instance, standardized bowel preparation scales, such as the Boston Bowel Preparation Scale (BBPS), have demonstrated that adequate cleaning significantly improves lesion detection during colonoscopy 4 , and suboptimal bowel preparation in patients undergoing early repeat colonoscopy is associated with lower adenoma detection rates 5 . Likewise, inadequate visualization in EGD may warrant further procedures, exposing patients to additional procedure risks and costs 6 . Unsurprisingly, leading gastroenterology organizations, including the American Society for Gastrointestinal Endoscopy (ASGE) and the British Society of Gastroenterology (BSG), have established that mucosal visualization is one of the essential quality indicators for EGD 6 7 .

Prior efforts to optimize visualization of the upper gastrointestinal tract during EGD include using cleaning agents such as simethicone, N-acetylcysteine, and pronase, with studies demonstrating improved endoscopist-rated visibility 8 9 10 . However, these studies have been limited by absence of validated cleanliness scoring systems. So far, several unvalidated scales have been employed to assess gastric and, in some cases, esophageal or duodenal visualization. Therefore, recent initiatives have attempted to establish validated tools for assessing mucosal cleanliness during EGD 11 12 . For instance, the Polprep scale, developed retrospectively across six Polish endoscopy units, demonstrated good intraobserver and interobserver reproducibility but was limited by its retrospective design 11 . More recently, the GRACE classification has been proposed as a practical tool for assessing mucosal visibility, although it also requires further external validation 12 . Contrastingly, the Toronto Upper Gastrointestinal Cleaning Score (TUGCS) was developed prospectively using Delphi methodology and validated in a clinical setting, offering a robust framework with high interrater reliability (0.79) and excellent test-retest reliability (0.83) 13 . The TUGCS evaluates mucosal visualization in four anatomical regions—fundus, body, antrum, and duodenum—using a scale from 0 to 3 per region: (0) presence of solid food, blood, clots, or unremovable content obstructing most of the area; (1) mucus, bubbles, liquid, or blood requiring suction and/or washing; (2) non-adherent liquid or blood requiring suction but no washing; and (3) entire mucosa clearly visible without suction or washing. Each region receives a numerical score (0–3), which is summed to yield a total score ranging from 0 (poor visibility) to 12 (excellent visibility). In its original study, the TUGCS showed significant correlation with global visualization ratings (r = 0.41, *P* = 0.002) 13 .

Although promising, the TUGCS has not been independently validated beyond its original study, nor has its relationship with lesion detection been thoroughly explored. Validating this tool could facilitate its widespread adoption, standardizing mucosal visualization assessment across clinical practice and research settings. This could enhance EGD quality, reduce the need for repeat procedures, and improve early lesion detection. This study aimed to validate the TUGCS as a reliable and practical tool for assessing mucosal cleanliness and visualization during EGD. It also investigated its correlation with lesion detection—an aspect not addressed in the original publication. We hypothesized that the TUGCS would exhibit adequate agreement between and within observers, addressing the question of its consistency in evaluating mucosal visualization quality.

## Patients and methods

### Study design and patients

This prospective, single-center study was conducted at the Instituto Nacional de Ciencias Médicas y Nutrición Salvador Zubirán (INCMNSZ), a tertiary care facility in Mexico City. Patients were enrolled from July 6, 2024, to December 18, 2024. Our Institutional Review Board (END-5129–24–25–1) approved the study.

We included patients aged ≥ 18 years who were undergoing diagnostic EGD for upper gastrointestinal symptoms (e.g., dyspepsia, reflux, anemia) or malignancy screening and for follow-up endoscopies. All patients provided informed consent. We excluded individuals who underwent EGD in the context of acute gastrointestinal bleeding, pregnancy, known esophageal, gastric, or duodenal cancer, or existing stenosis that hindered complete visualization of the upper gastrointestinal tract. Participants with incomplete endoscopic studies were excluded from the analysis.

### Equipment

We employed Olympus EVIS Exera 2 or EVIS X1 systems (GIF-H190; Olympus America, Center Valley, Pennsylvania, United States) to ensure high-quality imaging. Before each procedure, the endoscopic equipment was calibrated according to manufacturer guidelines to maintain consistency in image quality and visualization across all EGDs. Videos for the intraobserver subset were recorded using the Med X Change 4K and HD video recorder. Patient-identifying data were removed to maintain patient confidentiality and comply with ethical standards.

### Procedure and TUGCS application

Selected EGD procedures were performed and recorded by gastroenterology residents (non-experts) with at least 100 prior upper endoscopies, supervised by endoscopy fellows or attending endoscopists (experts) with over 500 upper endoscopies. Fourteen gastroenterology residents and seven attendings or advanced endoscopy fellows participated in the study. EGDs were conducted under moderate-to-deep sedation by an anesthesiologist using propofol-based anesthesia, ensuring patient comfort and procedure stability. Patients underwent a standard pre-procedure preparation, which included fasting for a minimum of 6 hours. Some patients had both EGD and colonoscopy as a double study, with no differences in fasting time; a 4-L polyethylene glycol (PEG) solution was administered the night before to prepare for colonoscopy, ensuring consistency in mucosal visualization for EGD. Simethicone (80 mg) was administered at endoscopist discretion, either 20 to 30 minutes before or during the procedure, to enhance mucosal visualization, and its use was recorded for subgroup analysis.

Two independent observers applied the TUGCS during each procedure: one gastroenterology resident (non-expert) and either an advanced endoscopy fellow or an attending endoscopist (experts). All physicians received a 5- to 10-minute training session before the study to standardize application of the TUGCS. Scores were recorded independently by both observers at procedure conclusion, blinded to each other’s responses. Videos were recorded during the initial procedures and randomly selected for intraobserver analysis. All patient-identifying data were removed and the videos were assigned a registration number for identification. Finally, these were reviewed at least 4 weeks after the EGD by the same physician using a standard video player. Physicians were instructed to assign a score to the video a second time after reassessment to evaluate intraobserver agreement.

Endoscopy rooms were equipped with small posters or reference sheets displaying the TUGCS, including illustrative pictures to assist observers during scoring if needed. At the end of each procedure, a subjective cleaning assessment was independently obtained from both observers, classifying mucosal visualization as inadequate, fair, good, very good, or excellent. Categories were defined as follows: inadequate (repeat EGD required; < 90% of mucosa visualized despite suction/washing), fair (solid/semi-solid/liquid with < 90% of mucosa visualized after suction/washing), good (semi-solid/liquid with > 90% of mucosa visualized), and excellent (minimal clear liquid with >95% of mucosa surface seen). Global mucosal visualization was also assessed subjectively using a classification adapted from the Aronchick bowel preparation scale and as applied in the original TUGCS validation study by Khan et al 13 .

Biopsies were performed at the discretion of the endoscopist for diagnostic or therapeutic purposes, without interference from the study protocol. Biopsies were typically targeted when endoscopic abnormalities were suspected (e.g., polyps, ulcers, mucosal irregularities), at the discretion of the attending endoscopist. Random biopsies were not mandated by the study protocol.

### Data collection

Data obtained included the TUGCS, comprising segmental scores (0–3) for each anatomical region (fundus, body, antrum, duodenum), and a total score (0–12). Biopsies were reviewed by three gastrointestinal pathologists, who identified *Helicobacter pylori* infection, intestinal metaplasia, dysplasia, and cancer. Specific polyp types (e.g., fundic gland, hyperplastic, neuroendocrine tumors, adenomas) were analyzed to identify premalignant or malignant lesions.

Additional data recorded included age, sex, indication for EGD, procedure duration, use of simethicone (and timing), fasting duration, global visualization rating (inadequate, fair, good, very good, excellent), and detection of lesions (presence, type, number, and anatomical location for endoscopic and histopathological findings, including types of polyps).

### Sample size

Sample size was calculated based on the original TUGCS validation study 13 , targeting an ICC of 0.79 compared with a reference value of 0.85, with α = 0.05 and power (1–β) = 0.8. The calculated sample size was 169 patients.

### Objectives

Our primary objective was to determine validity of the TUGCS through its interobserver and intraobserver agreement. Our secondary objective was to investigate the association between quality of mucosal visualization, as assessed by the TUGCS, and detection of specific lesions in the upper gastrointestinal tract. We reported both endoscopic and histopathological findings.

Primary endpoints included interobserver agreement, measured using the intraclass correlation coefficient (ICC) between TUGCS scores assigned by two independent observers during the procedure, and intraobserver agreement, assessed using the ICC for TUGCS segmental and total scores based on video reviews. An exploratory outcome examined the relationship between TUGCS scores and detection of upper gastrointestinal lesions, documented by type, number, and anatomical region.

### Statistical analysis

Descriptive statistics were employed to summarize participant characteristics. Categorical variables were presented as percentages, whereas continuous variables were expressed as means with standard deviations (SD) or medians with interquartile ranges, depending on data distribution (assessed using the Kolmogorov-Smirnov test). Interobserver and intraobserver agreements for TUGCS scores were evaluated using Cohen’s weighted kappa for segmental scores and ICC for total scores. Agreement levels were categorized as poor (< 0.4), fair (0.4–0.59), good (0.6–0.74) and excellent (0.75–1.0) for ICC, and slight (0.01–0.2), fair (0.21–0.4), moderate (0.41–0.6), substantial (0.61–0.8) and excellent (0.81–1.0) for Cohen’s kappa. Spearman’s rank correlation was utilized to analyze the relationship between TUGCS scores and subjective cleaning assessments. To determine the optimal cutoff for TUGCS in regard to endoscopic findings, we performed ROC analysis, which established an optimal cutoff of 6.5. Therefore, we finally performed univariate and multivariate logistic regression analysis to examine the association between high (6 or more) or low (5 or less) TUGCS scores and gastric and duodenal findings, adjusting for age, indication for EGD, and procedure duration, with odds ratios (ORs) and 95% confidence intervals (CIs) reported. *P* < 0.05 was regarded as statistically significant. Analyses were conducted using R software Version 2023.06.1+524.

## Results

### General findings

A total of 175 patients who underwent esophagogastroduodenoscopy were included in the study. Baseline characteristics are presented in Table 1 . Mean age of participants was 58.7 years (SD 15.3), with females accounting for 61% (106/175) and males for 39% (69/175). The most common indication for EGD was dyspepsia and gastroesophageal reflux disease (GERD), which both occurred in 15.4% (27/175) of patients each, followed by anemia/ferropenia at 13.7% (24/175), portal hypertension at 13.1% (23/175), dysphagia at 12.0% (21/175), and screening or follow-up for premalignant lesions (such as atrophic gastritis and metaplasia) at 11.4% (20/175). Other less common indications included follow-up of previous lesions at 5.7% (10/175), ruling out celiac disease in cases of chronic diarrhea and non-acute gastrointestinal bleeding both at 4.0% (7/175) each, Barrett’s esophagus at 3.4% (6/175), non-intentional weight loss at 1.1% (2/175), and baseline EGD before bariatric surgery at 0.5% (1/175). Mean fasting duration was 12.1 hours (SD = 2.6), and mean procedure duration was 12.0 minutes (SD 5.3). Simethicone was used in 19.4% of procedures (34/175), with 94% (32/34) receiving it prior to the procedure and 6% (2/32) receiving it during the procedure. Combined EGD and colonoscopy were performed in 14.9% of cases (26/175). Biopsies were performed in 93 of 175 patients (53.1%). The majority were targeted biopsies based on visual findings, although the exact breakdown of targeted versus non-targeted biopsies was not systematically recorded.

**Table:** **Table 1** Baseline patient characteristics.

Characteristics	Value
Total patients, n	175
Age, years, mean (SD)	58.7 ± 15.3
Gender, n (%)	
Male	69 (39%)
Female	106 (61%)
Fasting duration (hours); mean (SD)	12.1 ± 2.6
Procedure duration (min); mean (SD)	11.9 ± 5.3
EGD + colonoscopy. n (%)	26 (14.9%)
Simethicone Use, n (%)	
Yes	34 (19.4%)
During procedure	32 (18.3%)
Prior to the procedure	2 (1.1%)
EGD, esophagogastroduodenoscopy; SD, standard deviation.	

### TUGCS interrater findings

Interobserver agreement for the Toronto Upper Gastrointestinal Cleaning Score (TUGCS) was assessed using the ICC for total scores and Cohen’s weighted kappa for segmental scores. The ICC for the total TUGCS score was 0.95 (95% CI 0.93–0.96), indicating excellent agreement between gastroenterology residents and endoscopy fellows/attendings ( Table 2 ). The segmental interobserver agreement using weighted kappa (κ) also was excellent: fundus (κ = 0.86, 95% CI 0.81–0.92), body (κ = 0.90, 95% CI 0.86–0.94), antrum (κ = 0.92, 95% CI 0.89–0.96), and duodenum (κ = 0.92, 95% CI 0.88–0.96).

**Table:** **Table 2** Intraobserver and interobserver agreement for TUGCS scores by observer group.

Measure	Observer group	TUGCS total (ICC, 95% CI)	Subjective cleaning (κ, 95% CI)
Interobserver agreement	All observers	0.95 (0.93–0.96)*	0.889 (0.852–0.927)*
Intraobserver agreement	Non-experts (Residents)	0.896 (0.828 – 0.938)	0.779 (0.713–0.832)
	Experts (Fellows/Attendings)	0.924 (0.873–0.955)	0.776 (0.709–0.829)
Difference (z, P-value)	Non-experts vs. Experts	0.845, *P* = 0.398	0.026, *P* = 0.97
CI, confidence interval; ICC, intraclass correlation coefficient; κ = Kappa coefficient; TUGCS, Toronto Upper Gastrointestinal Cleaning Score. Agreement levels: poor (< 0.40), fair (0.40–0.59), good (0.60–0.79), excellent (> 0.80). The z-test compares Kappa or ICC differences between non-experts and experts, with P values indicating no significant differences. *Interobserver agreement is reported for all observers combined. TUGCS segmental ICCs (fundus, body, antrum, duodenum) are omitted for brevity.			

### TUGCS intrarater findings

Intraobserver agreement for TUGCS was assessed using 56 video recordings reviewed 4 weeks after the initial procedure, also employing intraclass correlation coefficient. For non-experts (gastroenterology residents), ICC for the total TUGCS score was 0.896 (95% CI 0.828–0.938), indicating excellent agreement. For experts (endoscopy fellows/attendings), intraobserver agreement for the total TUGCS score was 0.924 (95% CI 0.873–0.955), also indicating excellent agreement. A z-test revealed no significant difference in ICCs between non-experts and experts for total TUGCS scores (z = 0.845, *P* = 0.398). Results for both interrater and intrarater findings are presented in Table 2 .

### TUGCS with general mucosal visualization

Subjective cleaning assessments that classified mucosal visualization as inadequate, fair, good, very good, or excellent were provided by both observers at the end of each procedure. Procedures were rated by endoscopy experts as excellent (11%, 20/175), very good (35%, 62/175), or good (31%, 54/175), with fair ratings in 15% (27/175) and inadequate ratings in 7% (12/175) of cases. Conversely, gastroenterology residents reported preparation as excellent (12%, 21/175), very good (35%, 61/175), or good (26%, 45/175), with fair ratings in 20% (35/175) and inadequate ratings in 7% (13/175) of cases. The weighted *Kappa* coefficient for interobserver agreement on subjective ratings was 0.889 (95% CI 0.852–0.927), indicating excellent agreement.

**Fig. 1:**
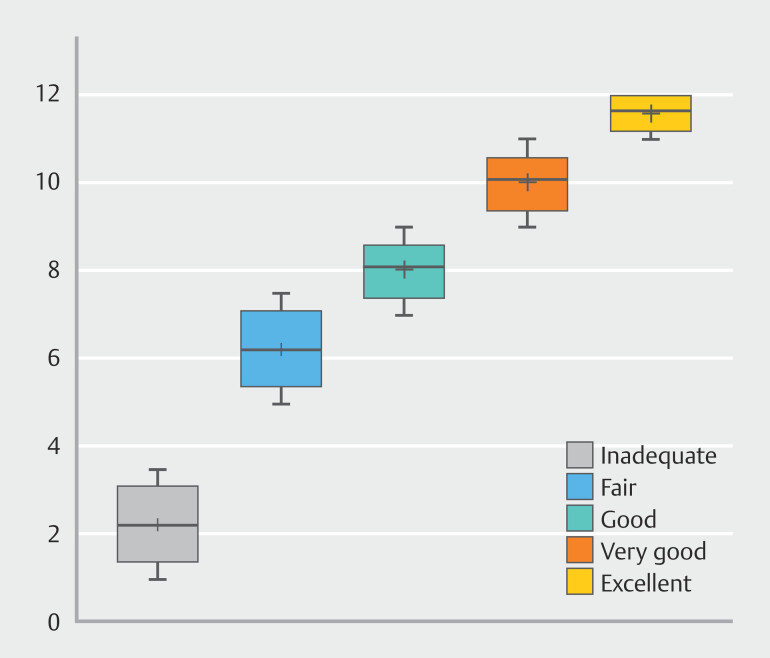
Median TUGCS scores by subjective cleaning assessments. Median scores and IQRs are based on total TUGCS scores (0–12) for each subjective cleaning category (inadequate, fair, good, very good, excellent) as assessed by both observers. Spearman’s rank correlation showed a significant positive correlation between TUGCS scores and subjective ratings (ρ = 0.68, *P* < 0.001).

Spearman’s rank correlation analysis demonstrated a strong positive correlation between TUGCS total scores and subjective cleaning assessments across gastroenterology residents (ρ = 0.779, 95% CI 0.713–0.832, *P* < 0.001) and endoscopists (ρ = 0.776, 95% CI 0.709–0.829, *P* < 0.001), indicating that higher TUGCS scores (indicating better mucosal visualization) were associated with better subjective ratings (e.g., excellent or very good). Median TUGCS scores by subjective cleaning assessments are presented in Fig. 1 , demonstrating a significant positive correlation between TUGCS and global ratings of mucosal visualization (ρ = 0.68, *P* < 0.001). According to expert endoscopists, mucosal visualization was rated as excellent in 11% (20/175), very good in 35% (62/175), good in 31% (54/175), fair in 15% (27/175), and inadequate in 7% (12/175). Gastroenterology residents reported similar proportions: excellent 12% (21/175), very good 35% (61/175), good 26% (45/175), fair 20% (35/175), and inadequate 7% (13/175).

Among the 175 patients, 152 (86.9%) achieved TUGCS total scores of 6 or more. Within the high-score group (TUGCS ≥ 6), 20 patients (13%) received an “excellent” subjective rating, 60 (39.7%) a “very good” rating, and 51 (33.6%) a “good” rating. Only 20 patients (13%) with a TUGCS ≥ 6 were rated as “fair” or “poor”. These distributions are consistent with a strong correlation between higher TUGCS and better subjective assessments of cleanliness.

### Association of simethicone use with TUGCS

An exploratory analysis revealed that simethicone use during the procedure was associated with lower TUGCS scores, suggesting that worse visualization may prompt intraprocedure simethicone administration OR 0.81 (95% CI 0.70–0.93), *P* = 0.002).

### Endoscopic and histopathological findings

Abnormal endoscopic findings in the stomach and duodenum were detected in 66.8% of procedures (117/175). Gastric findings included presence of polyps or tumors (n = 26), atrophic gastritis (n = 25), other unspecified gastropathies (n = 19), erosive gastropathy (n = 18), gastric ulcers (n = 17), intestinal metaplasia (n = 15), gastric ectasias (n = 3), one subepithelial lesion and one case of gastric varices. In regard to the duodenum, abnormal findings were detected in 35 procedures. The distribution of gastric and duodenal findings according to TUGCS score categories is shown in Table 3 . Duodenal findings included erosions (n = 18), polyps or tumors (n = 8), ulcers, (n = 4), villous atrophy (n = 2), duodenal ectasias (n = 2) and a case of a duodenal diverticulum. Table 4 compares frequency of findings in patients with a TUGCS ≤ 5 to those with ≥ 6. No statistically significant differences between individual findings were found.

**Table:** **Table 3** Frequency of endoscopic and histopathological findings in patients with TUGCS ≤ 5 compared with those with ≥ 6.

Endoscopic findings	Total (n = 175)	TUGCS ≤ 5 (n = 23)	TUGCS ≥ 6 (n = 152)	*P* value
Gastric findings				
Atrophic gastritis	25	1 (4%)	24 (15%)	0.253
Erosive gastropathy	18	2 (9%)	16 (11%)	0.787
Subepithelial lesions	1	0 (0%)	1 (0.6%)	N/A
Polyps or tumors	26	2 (9%)	24 (16%)	0.372
Intestinal metaplasia (probable)	15	1 (4%)	14 (9%)	0.603
Gastric ulcers	17	3 (13%)	14 (9%)	0.563
Gastropathy (unspecified)	19	1 (4%)	18 (12%)	0.282
Gastric varices	1	0 (0%)	1 (0.6%)	N/A
Gastric ectasias	3	0 (0%)	3 (2%)	N/A
Duodenal findings				
Duodenal erosions	18	2 (9%)	16 (11%)	0.788
Duodenal ulcers	4	1 (4%)	3 (2%)	0.478
Polyps or tumors	8	0 (0%)	8 (5%)	N/A
Villous atrophy	2	0 (0%)	2 (1%)	N/A
Duodenal diverticulum	1	0 (0%)	1 (0.6%)	N/A
Duodenal ectasias	2	1 (4%)	1 (0.6%)	0.121
TUGCS, Toronto Upper Gastrointestinal Cleaning Score.				

**Table:** **Table 4** Univariate and multivariate logistic regression for TUGCS (as a continuous variable and in TUGCS ≥ 6) as a predictor of gastroduodenal findings, adjusted for age, sex, procedure duration, and simethicone use.

Characteristics	OR (CI)	*P* value	aOR (CI)	*P* value
Male sex	0.986 (0.518–1.877)	0.966	TUGCS (cont): 1.078 (0.543–2.141)	0.830
			TUGCS ≥ 6: 1.190 (0.587–2.412)	0.630
Age	0.966 (0.976–1.017)	0.996	TUGCS (cont): 0.996 (0.975–1.018)	0.709
			TUGCS ≥ 6: 0.999 (0.977–1.022)	0.957
Procedure duration (minutes)	1.041 (0.977–1.110)	0.212	TUGCS (cont): 1.042 (0.979–1.109)	0.193
			TUGCS ≥ 6: 1.042 (0.979–1.110)	0.194
Simethicone use	1.239 (0.548–2.801)	0.607	TUGCS (cont): 1.595 (0.676–3.766)	0.287
			TUGCS ≥ 6: 1.277 (0.541–3.103)	0.576
TUGCS (cont)	1.164 (1.037–1.309)	0.010	TUGCS (cont): 1.192 (1.052–1.352)	0.006
			TUGCS ≥ 6: NA	
TUGCS > 6	4.753 (1.879–12.020)	< 0.001	TUGCS (cont): NA	< 0.001
			TUGCS ≥ 6: 5.152 (1.955–13.579)	< 0.001
aOR, adjusted odds ratio; CI, confidence interval; cont, continuous; OR, odds ratio; TUGCS, Toronto Upper Gastrointestinal Cleaning Score. *Endoscopic findings: gastric (polyps, ulcers, subepithelial lesions); duodenal (ulcers, polyps).				

### Association between endoscopic and histopathological findings and TUGCS

TUGCS scores as a continuous variable and TUGCS ≥ 6 were significantly associated with gastric or duodenal endoscopic and histologic findings. In the univariate logistic regression analysis, TUGCS as a continuous variable had an OR of 4.753 (1.879–12.020, *P* < 0.001), with similar findings after adjusting for age, sex, procedure duration, and simethicone use (adjusted OR [aOR] 1.192; 1.052–1.352, *P* = 0.006). Similarly, a TUGCS > 6 was associated with gastric or duodenal findings both in the univariate analysis (OR 4.753; 1.879–12.020, *P* < 0.001) and after adjusting for age, sex, procedure duration, and simethicone use (aOR 5.152; 1.955–13.579, *P* < 0.001).

## Discussion

This study validates the TUGCS as a reliable tool for assessing mucosal cleanliness and visualization during EGDs, demonstrating excellent interobserver and intraobserver agreement. Interobserver ICC for the total TUGCS score was 0.95 (95% CI 0.93–0.96), whereas intraobserver ICCs ranged from 0.896 (95% CI 0.828–0.938) among residents to 0.924 (95% CI 0.873–0.955) among experts. These values are even higher than those reported in the original Canadian validation study by Khan et al. (ICC = 0.79–0.83), possibly due to use of standardized reference posters in endoscopy rooms, a brief structured training session on TUGCS application, and greater familiarity driven by the higher number of scored procedures in this cohort 3 . The strong correlation between TUGCS and subjective cleaning assessments (ρ = 0.68, *P* < 0.001) further confirms its clinical utility in objectively quantifying mucosal visualization 13 . Although subjective global assessments also correlated strongly with TUGCS in our study, subjective impressions are inherently limited by interobserver variability and lack of standardization across centers. In contrast, TUGCS provides a structured, validated, and reproducible scoring system analogous to the BBPS in colonoscopy. This objectivity is critical for multicenter research, training, and quality improvement initiatives, where reproducibility and comparability are essential. Moreover, TUGCS yields a continuous score (0–12), enabling robust statistical associations with outcomes such as lesion detection, whereas subjective ratings remain descriptive and less precise.

This study provides support that cleanliness scores, such as TUGCS, are associated with lesion detection, including premalignant/malignant and endoscopic findings, mirroring evidence from colonoscopy studies where bowel preparation scores (e.g., BBPS) improve adenoma detection rates 5 14 15 . The association between TUGCS and these lesions highlights broader applicability of standardized visualization scores across endoscopic procedures. This reinforces their role in enhancing diagnostic accuracy, as noted in quality guidelines for both upper and lower gastrointestinal endoscopy 6 7 . In our study, detection of relevant pathology was significantly higher in procedures with TUGCS scores ≥ 6 (aOR 5.152; 95% CI 1.955–13.579; *P* < 0.001). Furthermore, higher TUGCS as a continuous variable was also associated with lesion detection (aOR 1.192; CI 1.052–1.352; *P* = 0.006.). Enhanced visualization with higher TUGCS likely helps detect subtle mucosal changes, which can aid in identifying lesions. Our results underscore TUGCS value for identifying at-risk patients, particularly those with lower TUGCS scores, which may lead to potentially premalignant missed lesions. This aligns with recommendations for enhanced endoscopic visualization, even in regions with moderate-prevalence settings of gastric malignancy 16 17 .

A notable limitation of the TUGCS is absence of a validated cutoff to determine adequacy or guide repeat endoscopy, unlike colonoscopy where a BBPS score ≥ 6 is commonly used. In our study, a threshold ≥ 6 was applied as an exploratory analysis based on a ROC curve that identified 6.5 as the optimal point, which aligns similarly with the ≥ 6 benchmark already established in colonoscopy. Nevertheless, this cutoff should be interpreted cautiously, because a total score of 6 can result from different segmental combinations (e.g., excellent antrum/duodenum but poor fundus/body) which may not uniformly represent adequate visualization. Lack of a standardized threshold currently limits immediate clinical applicability of TUGCS. Future multicenter studies are needed to validate whether total or segmental minimum scores can standardize clinical decision-making and reduce unnecessary repeat procedures.

A key strength of this study is its prospective design and a large sample size of 175 patients, providing robust statistical power to evaluate TUGCS reliability. Notably, TUGCS proved equally valuable for both trainees and experts, with comparable inter and intraobserver agreement, highlighting its utility as a standardized tool across all training levels. This finding addresses a gap in prior TUGCS validation, which included only expert endoscopists. Use of high-definition endoscopy systems and standardized training for TUGCS scoring further strengthens reproducibility, a critical factor for endoscopic quality metrics. Our multivariate analysis, adjusted for indication, procedure duration, and age, offers a comprehensive assessment of TUGCS predictive value, providing novel insights into its possible role in detecting clinically relevant lesions.

Some limitations warrant consideration. First, the single-center design limits generalizability to other populations or healthcare settings with different endoscopic practices or demographics and our cohort had a predominance of women, which reflects the referral pattern of our center as a tertiary institution for autoimmune diseases. Multicenter validation would strengthen TUGCS broader applicability. Second, the low rate of use of pre-procedure antifoaming agents (simethicone) may introduce bias, potentially underestimating its benefits on TUGCS scores and lesion detection. Third, use of two different endoscopy processors (Olympus EVIS Exera 2 and EVIS X1) could theoretically introduce variability in mucosal visualization. However, consistency of TUGCS reliability across both systems suggests robustness of the tool independent of imaging platform, thereby supporting its potential applicability across centers with different equipment. Finally, relying on random videos for intraobserver analysis, without specifying the subset, may introduce selection bias, although blinding and independence mitigate this risk.

Future research should focus on multicenter validation of TUGCS across diverse populations, longitudinal studies examining its impact on patient outcomes, including the progression of precancerous lesions or cancer detection, and investigations into optimal TUGCS thresholds for repeat EGD.

## Conclusions

In summary, adopting TUGCS could have the potential to revolutionize the landscape of EGD practices and significantly enhance patient outcomes.
